# Augmentation of Clozapine in Treatment Resistant Schizophrenia – Is There a Superior Strategy?

**DOI:** 10.1192/bjo.2025.10139

**Published:** 2025-06-20

**Authors:** Priya Babla, Declan Hyland

**Affiliations:** ^1^University of Liverpool, Liverpool, United Kingdom; ^2^Mersey Care NHS Foundation Trust, Liverpool, United Kingdom

## Abstract

**Aims:** Schizophrenia affects approximately 20 million people worldwide, with 30% of cases being classified as treatment resistant schizophrenia (TRS) cases. Clozapine remains the gold standard for managing TRS, but around 40% of patients treated with clozapine fail to demonstrate optimal treatment response. This literature review aims to evaluate various augmentation strategies used to enhance clozapine’s effectiveness for TRS, assessing data from systematic reviews and meta-analyses published between 2010 and 2024, the aim of which is to identify whether there is a consensus surrounding augmentation strategies used, whilst simultaneously assessing the quality of the existing research, and recommending where further research could guide future clinical practice and prescribing in TRS.

**Methods:** A systematic search was conducted in February 2024 across Ovid and PubMed databases using the keywords “schizophrenia”, “clozapine”, and “augmentation”. Inclusion criteria were: studies focusing on clozapine augmentation with another pharmacological agent, systematic reviews or meta-analyses published between 2010 and 2024, and studies from credible peer-reviewed sources. Case reports, single-agent studies, and non-peer-reviewed sources were excluded. A total of 181 papers were screened and, after applying inclusion and exclusion criteria, three systematic reviews were selected for analysis. The quality of each study was assessed using the CASP checklist.

**Results:** Grover et al. (2022) concluded that augmentation with antipsychotics (specifically aripiprazole) and Electroconvulsive Therapy (ECT) provided the most promising results. Siskind et al. (2018) supported the use of aripiprazole, fluoxetine, sodium valproate, and memantine for overall psychosis symptoms, while memantine was highlighted for its use in negative symptoms of schizophrenia. Porcelli et al. (2011) emphasised that several strategies showed promise, but methodological limitations prevented firm recommendations being made. Aripiprazole and ECT were highlighted, but only tentatively given the sample sizes and potential biases. Across all reviews, aripiprazole was identified as an effective pharmacological augmentation agent option, with ECT also emerging as a beneficial adjunct. However, many trials were repeated across the reviews, which may introduce bias and skew conclusions, limiting their relevance within the broader treatment realms.

**Conclusion:** This review establishes a need for more rigorous longitudinal studies to determine the most effective augmentation strategies for clozapine in TRS patients. The evidence remains inconclusive due to methodological issues like small sample sizes and study heterogeneity. Future research should prioritise comparative efficacy trials, long-term follow-ups, and increased participant diversity – especially addressing under-representation of women in schizophrenia research. These efforts are essential to improving treatment outcomes for TRS patients who remain symptomatic despite being on clozapine.

